# Enterovirus D68–Associated Acute Respiratory Illness — New Vaccine Surveillance Network, United States, July–October, 2017 and 2018

**DOI:** 10.15585/mmwr.mm6812a1

**Published:** 2019-03-29

**Authors:** Stephanie A. Kujawski, Claire M. Midgley, Brian Rha, Joana Y. Lively, W. Allan Nix, Aaron T. Curns, Daniel C. Payne, Janet A. Englund, Julie A. Boom, John V. Williams, Geoffrey A. Weinberg, Mary A. Staat, Rangaraj Selvarangan, Natasha B. Halasa, Eileen J. Klein, Leila C. Sahni, Marian G. Michaels, Lynne Shelley, Monica McNeal, Christopher J. Harrison, Laura S. Stewart, Adriana S. Lopez, Janell A. Routh, Manisha Patel, M. Steven Oberste, John T. Watson, Susan I. Gerber

**Affiliations:** ^1^Epidemic Intelligence Service, CDC; ^2^Division of Viral Diseases, National Center for Immunization and Respiratory Diseases, CDC; ^3^IHRC, Atlanta, GA; ^4^Seattle Children’s Hospital, Seattle, Washington; ^5^Texas Children’s Hospital, Houston, Texas; ^6^Baylor College of Medicine, Houston, Texas; ^7^UPMC Children’s Hospital of Pittsburgh, Pittsburgh, Pennsylvania; ^8^University of Rochester School of Medicine and Dentistry, Rochester, New York; ^9^Department of Pediatrics, University of Cincinnati College of Medicine, Division of Infectious Diseases, Cincinnati Children’s Hospital Medical Center, Cincinnati, Ohio; ^10^The Children’s Mercy Hospital, Kansas City, Missouri; ^11^Vanderbilt University Medical Center, Nashville, Tennessee.

In the fall of 2014, an outbreak of enterovirus D68 (EV-D68)–associated acute respiratory illness (ARI) occurred in the United States (*1*,*2*); before 2014, EV-D68 was rarely reported to CDC (*2*,*3*). In the United States, reported EV-D68 detections typically peak during late summer and early fall (*3*). EV-D68 epidemiology is not fully understood because testing in clinical settings seldom has been available and detections are not notifiable to CDC. To better understand EV-D68 epidemiology, CDC recently established active, prospective EV-D68 surveillance among pediatric patients at seven U.S. medical centers through the New Vaccine Surveillance Network (NVSN) (*4*). This report details a preliminary characterization of EV-D68 testing and detections among emergency department (ED) and hospitalized patients with ARI at all NVSN sites during July 1–October 31, 2017, and the same period in 2018. Among patients with ARI who were tested, EV-D68 was detected in two patients (0.8%) in 2017 and 358 (13.9%) in 2018. Continued active, prospective surveillance of EV-D68–associated ARI is needed to better understand EV-D68 epidemiology in the United States.

NVSN conducts active, prospective, population-based surveillance for ARI* among children and teens aged <18 years at seven U.S. medical centers at Cincinnati, Ohio; Houston, Texas; Kansas City, Missouri; Nashville, Tennessee; Pittsburgh, Pennsylvania; Rochester, New York; and Seattle, Washington (*4*). Respiratory specimens (mid-turbinate nasal, oropharyngeal swabs, or both) from patients with ARI were tested at each site for EV-D68 using a validated real-time reverse transcription–polymerase chain reaction assay. Two NVSN sites (Nashville and Pittsburgh) tested all ARI specimens for EV-D68 directly. Five sites (Cincinnati, Houston, Kansas City, Rochester, and Seattle) used a two-step algorithm, wherein all ARI specimens were first tested for enterovirus/rhinovirus (EV/RV) using molecular diagnostic assays approved by the Food and Drug Administration or CDC; all EV-positive or RV-positive specimens were subsequently tested for EV-D68. Demographic and admission status information were collected from medical charts. EV-D68 detections were analyzed by year, month, site, admission status, and patient sex and age.

Based on preliminary data, test results were positive for EV-D68 for two (0.08%) of 2,433 patients with ARI who were tested during 2017 and 358 (13.9%) of 2,579 tested during 2018. In 2017, one patient whose test result was positive for EV-D68 was hospitalized in Houston, and one was evaluated in the ED in Rochester. In 2018, patients with EV-D68-positive test results were identified at all seven sites, and 242 of 358 patients (67.6%) were hospitalized (range by site = 53.3%–76.8%) (Table). EV-D68 was detected in 9.2% of patients with ED visits for ARI and 18.3% of hospitalized patients with ARI. Approximately half (169; 47.2%) of the 2018 EV-D68 detections occurred in September (Figure). The peak of detections varied by site, with Cincinnati and Kansas City peaking in late August through September; Houston, Pittsburgh, and Rochester in mid-September; and Nashville and Seattle in October. The median age of patients testing positive for EV-D68 was 3 years (range = 1 month–17 years; interquartile range = 1.5–5 years), and 211 (58.9%) were male. Among 42 EV-D68–positive specimens from 2018 sequenced at CDC, all were lineage B3.

**TABLE:** Number of patients with acute respiratory illness (ARI) who were tested and received results positive for EV-D68, by admission status and network surveillance site — New Vaccine Surveillance Network (NVSN), United States, July 1–October 31, 2018

Admission status/NVSN site	No. of ARI patients tested	No. (%) of EV/RV-positive patients	EV-D68-positive patients		
			No. of patients	% Among EV/RV-positive patients	% Among ARI patients tested
**Emergency department visit**					
Cincinnati	148	40 (27.0)	13	32.5	8.8
Houston	157	58 (36.9)	9	15.5	5.7
Kansas City	306	163 (53.3)	21	12.9	6.9
Nashville	282	N/A	21	N/A	7.4
Pittsburgh	198	N/A	25	N/A	12.6
Rochester	61	34 (55.7)	18	52.9	29.5
Seattle	104	73 (70.2)	9	12.3	8.7
All sites	1,256	368 (47.4)*	116	19.0^†^	9.2
**Inpatient**					
Cincinnati	235	102 (43.4)	43	42.2	18.3
Houston	220	62 (28.2)	16	25.8	7.3
Kansas City	139	92 (66.2)	33	35.9	23.7
Nashville	202	N/A	24	N/A	11.9
Pittsburgh	269	N/A	70	N/A	26.0
Rochester	161	108 (67.1)	45	41.7	28.0
Seattle	97	58 (59.8)	11	19.0	11.3
All sites	1,323	422 (49.5)*	242	35.1^†^	18.3
**Total**	**2,579**	790 (48.5)*	**358**	**27.6^†^**	13.9

**Abbreviations:** EV = enterovirus; N/A = not applicable; RV = rhinovirus.

* These percentages only include ARI patients at sites that first tested for EV/RV (Cincinnati, Houston, Kansas City, Seattle, and Rochester). For comparison, in 2017, a total of 715 of 1,714 (41.7%) ARI patients first tested positive for EV/RV.

^†^ These percentages only include EV-D68-positive patients at sites that first tested for EV/RV (Cincinnati, Houston, Kansas City, Seattle, and Rochester. For comparison, in 2017, two of 715 (0.3%) EV/RV-positive patients tested positive for EV-D68.

**FIGURE:**
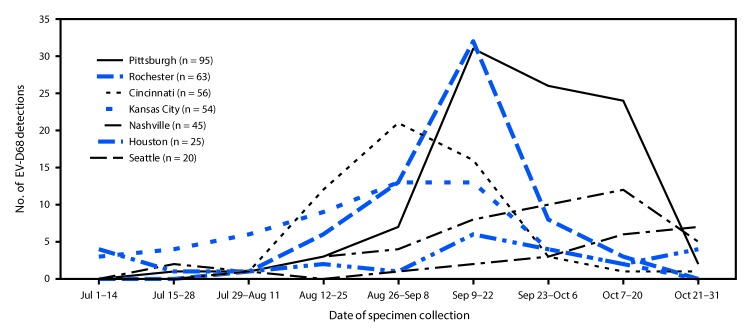
Enterovirus-D68 (EV-D68) detections, by date of specimen collection and surveillance network site (N = 358) — New Vaccine Surveillance Network, United States, July 1—October 31, 2018

## Discussion

During 2018, ARI surveillance through NVSN detected EV-D68 at levels substantially higher than those during the same period in 2017. European countries also reported EV-D68 activity in 2018 (*5*,*6*). Although EV-D68 infection more commonly causes respiratory illness, previous investigations have suggested that EV-D68 might also be associated with acute flaccid myelitis (AFM), a rare neurologic condition characterized by acute flaccid limb weakness (*7*,*8*). Contemporaneous with the 2014 outbreak of EV-D68 associated with respiratory illness, CDC received increased reports of AFM, supporting a temporal association between EV-D68 and AFM (*7*). Since 2015, CDC has conducted surveillance for AFM in the United States using a standardized case definition (*9*). As of March 1, 2019, CDC has confirmed 223 AFM cases in 2018, with peak onset of limb weakness in September 2018; in 2017, CDC confirmed 35 cases (*8*,*10*). Although AFM is rare in the United States, these AFM surveillance data, along with the EV-D68 activity documented through NVSN, provide additional supporting evidence for a temporal association between EV-D68 respiratory illness and AFM. CDC, in collaboration with clinical and public health partners, continues to investigate the relationship between AFM and enteroviruses, including EV-D68.

The findings in this report are subject to at least two limitations. First, this report describes EV-D68 testing within NVSN during July–October of each year, but additional cases likely occurred outside this period in 2018. Therefore, the results might not be representative of the entire EV-D68 season. Second, NVSN sentinel surveillance sites are geographically varied, but might not be representative of all regions of the United States.

Through recently established active, prospective, ARI surveillance in NVSN, EV-D68 was detected in 0.8% of patients tested in 2017 and 13.9% in 2018. Continued surveillance for EV-D68–associated ARI is needed to better understand the epidemiology of EV-D68 in the United States.

**Summary**

**What is already known about this topic?**

A nationwide outbreak of enterovirus D68 (EV-D68), which is associated with acute respiratory illness (ARI), occurred in 2014. EV-D68 epidemiology is not fully understood because testing in clinical settings is limited and detections are not notifiable to CDC.

**What is added by this report?**

Based on active, prospective surveillance of ARI through the New Vaccine Surveillance Network, EV-D68 was detected in two (0.8%) patients in 2017 and 358 (13.9%) in 2018. Detections in 2018 peaked in September.

**What are the implications for public health practice?**

Continued active, prospective surveillance is needed to better understand trends in EV-D68 circulation.

## Footnotes

* A child (aged <18 years) was eligible for inclusion in NVSN ARI surveillance if he or she visited the emergency department or were admitted to the hospital in the 48 hours preceding enrollment, with one or more of the following symptoms/events: fever; cough; earache; nasal congestion; runny nose; sore throat; vomiting after coughing; wheezing; shortness of breath/rapid or shallow breathing; apnea; apparent life-threatening event or brief resolved unexplained event; or myalgias and the duration of illness at the time of enrollment was <14 days. Children with a known nonrespiratory cause for hospitalization/ED visit were excluded from enrollment, as were children residing outside the surveillance area.
