# *Erratum:* Vol. 68, No. 12

**DOI:** 10.15585/mmwr.mm6815a6

**Published:** 2019-04-19

In the report “Enterovirus D68–Associated Acute Respiratory Illness — New Vaccine Surveillance Network, United States, July–October, 2017 and 2018,” a percentage was misreported in multiple places. On page 277, the sixth sentence of the first paragraph should have read “Among patients with ARI who were tested, EV-D68 was detected in two patients (**0.08%**) in 2017 and 358 (13.9%) in 2018.”

On page 278, in the second section of the summary box, the first sentence should have read “Based on active, prospective surveillance of ARI through the New Vaccine Surveillance Network, EV-D68 was detected in two (**0.08%**) patients in 2017 and 358 (13.9%) in 2018.”

On page 280, the first sentence of the last paragraph should have read “Through recently established active, prospective, ARI surveillance in NVSN, EV-D68 was detected in **0.08%** of patients tested in 2017 and 13.9% in 2018.”

